# Length of Hospital Stay and Bed Occupancy Rates in Former Yugoslav Republics 1989–2015

**DOI:** 10.3389/fphar.2016.00417

**Published:** 2016-11-07

**Authors:** Aleksandar Cvetkovic, Danijela Cvetkovic, Vladislava Stojic, Nebojsa Zdravkovic

**Affiliations:** ^1^Surgery Department, Faculty of Medical Sciences, University of KragujevacKragujevac, Serbia; ^2^Surgery Clinic, Clinical Centre KragujevacKragujevac, Serbia; ^3^Faculty of Science, Institute of Biology and Ecology, University of KragujevacKragujevac, Serbia; ^4^Department of Medical Statistics and Informatics, Faculty of Medical Sciences, University of KragujevacKragujevac, Serbia

**Keywords:** hospital care, healthcare globalization, transition, economic burden, healthcare cost

## Hospital services provision and financing legacy of former Yugoslavia

Recent decades have been witnesses that developing world economies preceded global economic growth with all the consequences on healthcare systems of their countries. Complex and dynamic socioeconomic and technological evolution, primarily in free market of capitalist economies as well as in former socialist countries/economies with some postponement, provided significant advances and improvements in health outcomes (Jakovljevic and Ogura, 2016). Large differences in the quality of health systems are evident. Globalization among many changes led to the creation of so called emerging markets. The one with the most intensive development were marked by Goldman Sachs as BRICS (Brazil, Russia, India, China, and South Africa; Jakovljevic, 2015).

European region offers great opportunities for studying variations in the magnitude of inequalities in health system quality because of the numerous intercountry varieties of political, cultural, economic, and epidemiologic nature (Arcaya et al., 2015). Eastern Europe and Balkans allow very good insight whether states with history of recent period of war, political instability, economic crisis, and different types of health care reforms have larger inequalities in health than countries elsewhere in Europe and worldwide (Mackenbach et al., 2008).

We conducted a study within former Yugoslav republics, today independent countries and tried to identify some of the indicators determining these variations. A shortage of published data related to the consequences of South-Eastern European transition on healthcare is evident, especially regarding former Yugoslavia. The civil war events between 1989 and 1995 initiated a huge wave of socioeconomic changes in the whole region of the Balkans that inevitably influenced national health systems quality referring to health care provision, planning and financing. These reforms were significantly more prosperous in some states of former Yugoslavia compared to the others (Jakovljevic et al., 2015a). National health system of former Yugoslavia was actually something in between Western European and Eastern Soviet bloc financing pattern. Serbia is the largest Western Balkans medical equipment and pharmaceutical market in terms of population size as well as the value of sales among neighboring non-EU countries. Global recession caused serious problems in provision of sustainable financing and increased shortages of pharmaceuticals and medical equipment across the region of former Yugoslavia (Jakovljevic et al., 2015b).

Health insurance systems are similar in all former Yugoslav republics. All employed citizens are insured and they use health care services in public health care institutions as well as in private institutions which have a signed contract with the Health Insurance Fund, a legal entity established by the Republic Ministry of Health. Unemployed citizens are insured by the employed family member premiums. One of the main functions of the Health Insurance Fund is collection of contributions for health insurance and contracting of health services. Health Insurance Fund cooperates closely with Pension and Disability Insurance Fund (Petrusic and Jakovljevic, 2015).

World-widely, out-of-pocket (OOP) payment for health care is the predominant form of health financing. The catastrophic expenditure is defined as an expenditure of over 40% of non-food household expenditure or 10% of overall household expenditure. Little is known about global distribution of catastrophic expenditure especially in developing countries (Shrime et al., 2015).

## The data report methods

### Public data set description—European health for all—HFA-DB

Public data sources used in this Data Report was the European Health for all Database (WHO, 2015) relying on: http://data.euro.who.int/hfadb/. HFA-DB provides health statistics data related to basic demographics, health status, risk factors, health-care resources, utilization, and expenditure in the 53 countries in the WHO European Region. The indicators are organized into following groups: demographic and socioeconomic statistics, mortality-based indicators, morbidity, disability, and hospital discharges, lifestyles, environment, health care resources health care utilization, and costs, maternal, and child health. It allows queries for country, intercountry, and regional analyses, and displays the results in tables, graphs, or maps, which can be exported for further use. The data are compiled from various sources, including a network of country experts, WHO/Europe's technical programs, and partner organizations, such as agencies of the United Nations system, the statistical office of the European Union (EUROSTAT) and the Organization for Economic Cooperation and Development.

Filters that we applied to these extensive data sources were: inpatient surgical procedures per year per 100 000, total number of inpatient surgical procedures per year, average length of stay for all hospitals, average length of stay for acute care hospitals only, bed occupancy rate (%) for acute care hospitals only and outpatient contacts per person per year. Although HFA-DB is updated twice a year, data availability can vary between countries, and indicators. We used the database for comparison and assessing the health care quality and trends in former Yugoslav republics (Slovenia, Croatia, Bosnia, and Herzegovina, Serbia, Montenegro, and FJR Macedonia) in an international context from 1989 to 2015. Data were acquired based on reported values to the WHO by the national officially released in respective years. Readers are free to access and reuse these publicly available data at the link provided (WHO, 2015).

## Impact of transitional health reforms to the regional hospital sector

Obviously, there are numerous differences in the quality of health services among the countries of the former Yugoslavia. Quality of health services can be expressed by parameters such as: inpatient surgical procedures per year per 100 000, total number of inpatient surgical procedures per year, average length of stay, average length of stay (acute care hospitals only), bed occupancy rate (%), (acute care hospitals only), and outpatient contacts per person per year, which all are the indicators studied in this paper. Today many interventional medical procedures are being performed in a modern way, with modern diagnostic and therapeutic equipment. It certainly contributes to faster and more efficient treatment, as well as the rapid return of patients in the living and working environment with all its economic implications (Caruso et al., 2016).

The significance of surgical disease worldwide has only recently been estimated. Although original estimations suggested that up to 11% of global morbidity and mortality is in correlation with surgical disease, more recent reports suggested that this number is much greater and that even up to 1/3 of the global burden of disease is surgical in nature (Shrime et al., 2016). Although improvements in regular open surgery are evident, laparoscopic surgery represents one of the most important breakthroughs in surgical treatment during recent years. It has many advantages such as shorter hospital stay, reduced pharmaceutics application, rare wound infections, etc., Thanks to modern advances today there is the concept of 1-day surgery (Zhou et al., 2016). However, minimally invasive approach also has a few disadvantages and one of the most important is limited range of motion. It has been eliminated by development of robotic surgery, which will further improve outcome, and reduce the length of hospital stay. Advantages of robotic surgery are evident, despite the high cost of use of such sophisticated equipment (Brunaud et al., 2011).

A short stay in hospital has its many advantages. First of all, the patient spends less time in an environment where they can become a victim of hospital infections that are very difficult to treat. This is particularly important for patients who require treatment in intensive care units. Resistance can occur in all types of pathogen agents (Kramer and Zimmerman, 2010; Barnett et al., 2013; Sganga et al., 2016).

**Figure 1:**
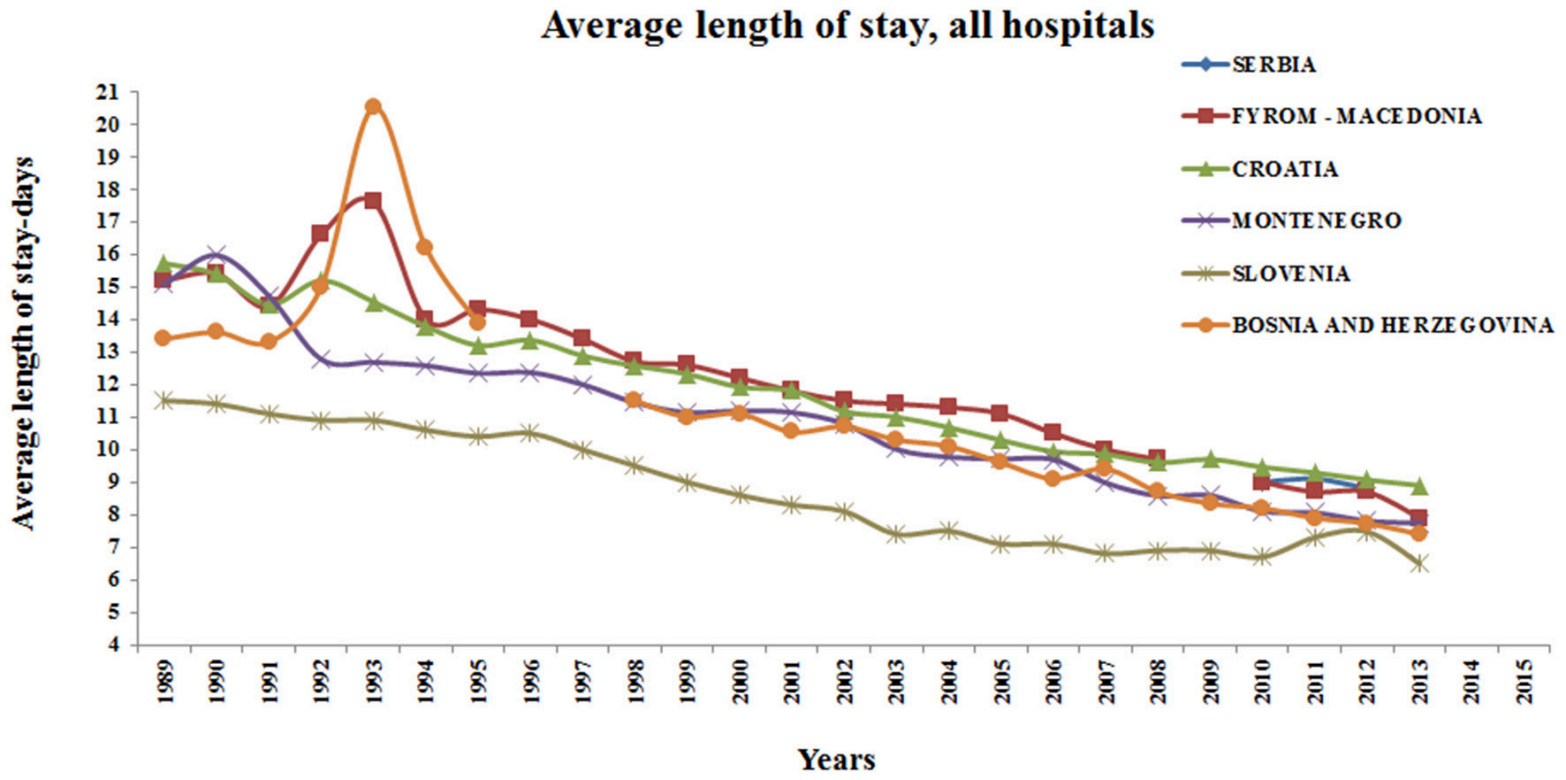
**Average length of stay, all hospitals, former Yugoslav republics**.

This trend of reducing the average length of hospital stay is present in all countries of the former Yugoslav states. In the reporting period, chart of the average length of stay in hospitals has the linear flow and displays a constant reduction. This is an expected result considering the penetration and adoption of new technical developments in the region, albeit with a slight delay compared to the developed parts of the world. This trend is most distinct in Slovenia. The explanation is probably in the fastest economic development of this country, which resulted in the use of modern means of treatment and medical equipment. For example, Slovenia is the first among former Yugoslav republics that included robotic surgery in routine practice using the da Vinci surgical system. Furthermore, Slovenia is the first of all the member states of the former Yugoslavia that entered the EU. Bosnia and Herzegovina has made great efforts to introduce the guides of good clinical practice, which involves reducing the misuse of antibiotics. That reduced the number of nosocomial infections and consequently length of hospital stay (Petrusic and Jakovljevic, 2015). Although the same trends can also be observed in Serbia, FJRM, Montenegro, and Croatia, their progress was not as impressive as in Slovenia. A fluctuation in the trend of shortening the average hospital stay, as the consequence of the civil war, is present in the period from 1990 to 1995. This trend is most prominent in Bosnia and Herzegovina and FJRM with a significant increase in the average hospital stay within the specified period. Slovenia had the shortest average length of stay for all hospitals, in regard to Croatia and Former Yugoslav Republic of Macedonia (Figure 1).

The economic aspect of the length of hospital stay should not be neglected, because the shortening of hospital stay leads to less consumption of often very expensive drugs, less engagement and fatigue of staff as well as to a faster return of patient to work place (Yin et al., 2013). The development of medicine leads to shortening of hospital stay, but nowadays, there are patients in very serious medical condition carried in intensive care units that previously would not have survived without new medical advances. It leads to an increase in the number of patients and duration of treatment (Karam et al., 2016). This case is especially obvious in intensive care units, where hospital infections are common problem. Failure to follow the rule of fast and well-timed discharge from clinic and misuse of pharmaceuticals lead to longer application of antibiotics, which can cause the development of multiresistance on antibiotics with well-known consequences. Frequent consequence of such manners in the administration of the antibiotics, especially in combination with proton pump inhibitors, is the emergence of Clostridium difficile colitis (Burnham and Carroll, 2013; Mathur et al., 2014; Kandel et al., 2016).

There are many medical breakthroughs that were previously available to a small number of patients or major medical centers. Also, human population constantly rises so that the number of newly diagnosed patients each year increases. The aging of population is also constant, with its entire spectrum of geriatric diagnosis burden. According to the generally accepted forecasts scenario, population aging will even accelerate its trend. Deep demographic transformation of modern societies started a century and a half ago in most of the developed states, but now, this phenomenon is moving from rich north hemisphere to the emerging markets of the south. Developing nations age even faster than developed part of the world. For example, increasing of the number of people over 60 years from 7 to 14% in France occurred over 115 years, while in China during only 26 years. So far, most of the global aging has occurred in the most developed regions of the world (UN, World Population Ageing, 2013).

**Figure 2:**
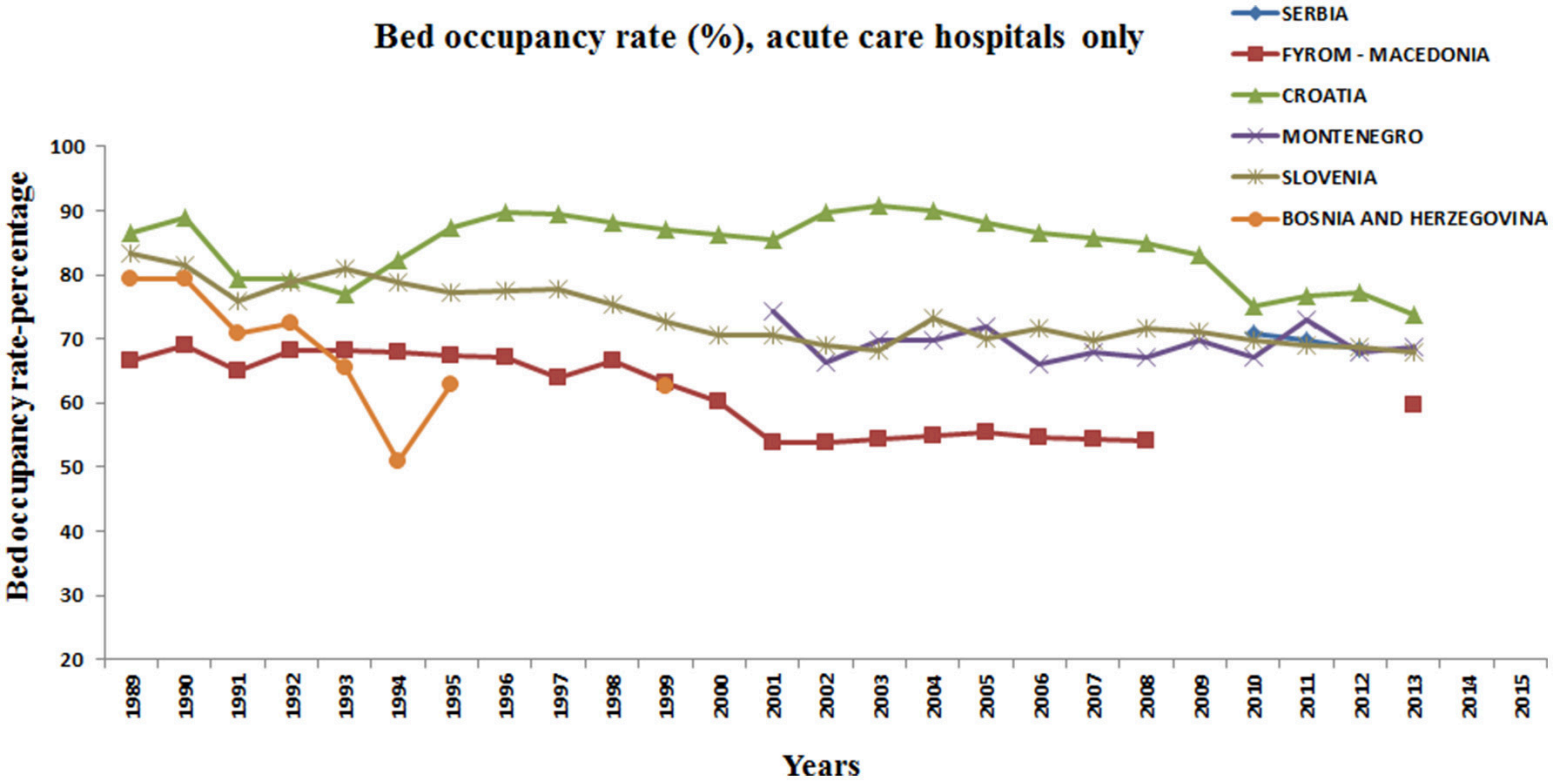
**Bed occupancy rate (%), acute care hospitals only, former Yugoslav republics**.

The development of diagnostics led to better detection of conditions and diseases; therefore today we have “incidentalomas” as incidentally discovered disease, which also requires time and money for medical treatment as well as the engagement of professional staff (Ye et al., 2016). Technical development with better diagnostics ant treatment caused extended longevity and together contributed to increasing in outpatient contacts per person per year in every former Yugoslav republic. The elderly citizens have higher medical needs (Getzen, 1992). Often last years of someone's life in an severe illness require expensive intensive care treatment or involvement of personnel home care service, with costs greater than over the entire lifespan (Kovacević et al., 2015). This trend is especially conspicuous in Croatia. Again, decrease of this trend observed in almost all studied countries from 1990 to 1995 can be attributed to civil war activities. Also, the same factors together with severe hospital infections led to increasing of bed occupancy rate in intensive care hospital units (Udy et al., 2013; Denny et al., 2016; Vincent et al., 2016). The same trend can be observed in all former Yugoslav republics. There is no decreasing in bed occupancy rate in acute care units during observed period from 1989 to 2015, despite the global shortening of average hospital stay (Figure 2).

Despite a great reduction of bed number due to cost saving measure, bed occupancy rate remained constant (Forster et al., 2003). Percentage of bed occupancy rate for acute care hospitals only has the highest level in Croatia and the lowest in Former Yugoslav Republic of Macedonia. Data for Montenegro until 2001 are missing.

The global health system changes in the former Yugoslavia must be viewed from the aspect of the relatively recent war events as well as the late Serbian economic opening to the world after 2000. During the war events there was an evident increase in the number of hospitalized patients in the field of trauma, with the consequences of treatment that are correlated with the degree of availability and quality of medical services in such circumstances. Slovenia and Croatia accomplished faster progress in the development of the health system services which is a trend that existed even before the war. The impacts of world macroeconomic crisis that started in 2008 should not be neglected. It pushed all of the Western Balkan countries into numerous difficulties in providing healthcare financing with all consequences in healthcare quality (Jakovljevic et al., 2016).

## Limitation of study

HFA-DB is updated twice a year, but data for some countries and some periods are missing. The data are collected from various local sources, which were insufficient in the former Yugoslav republics. It can be attributed to unfavorable socio-economic and political situation during recent years. The authors considered that this lack of data would not change the general trends landscape.

## Upcoming challenges for the hospital care in Western Balkans

The South Eastern Europe and Balkan represent numerous and very diverse ethnic, religious, and traditional patterns, lifestyle models, and social-economic strength levels (Jakovljevic, 2013). It is very important to provide a comprehensive insight into the population aging in this part of Europe, with all social and economic consequences on regional health systems development. However, there is a significant absence of population aging data and its consequences in the Eastern Europe and the Balkan (Jakovljevic and Laaser, 2015). Health policy makers in many developing countries are conscious that their national financial capabilities to invest in the most effective and also expensive medical procedures and drugs remain very limited. Resource distribution based on knowledge must be main determinant in health policy of developed and especially developing economies (Jakovljevic, 2013).

Final judgment on health care system quality is of course in the hands of patients, with easily identifiable parameters such as extended lifespan, fall in neonatal and maternal mortality, and decreasing rates of communicable diseases. Life expectancy at birth and good health at an advanced age are much higher in high-income countries with leading of Japan (Ogura and Jakovljevic, 2014). Accelerated population aging will affect nations in demographic transition much more compared to developed economies (Jakovljevic, 2015). Financial performance of each of the local health care system must be based on local particularities. Health insurance systems in countries of South Eastern Europe must be reformed based on the insurance models of the leading countries but certainly in the light of the peculiarities of each domestic economy. Unfortunately, simple copying of health system from developed countries is not applicable for developing economies due to their substantially different legacy, traditional value systems and socioeconomic status (Jakovljević et al., 2011). One way to concretize the impact of disease, global or local, is to estimate the economic consequences it causes. Although there is a debate in published data regarding how health and economic status are connected, it is evident that good global population health contributes to economic prosperity (Alkire et al., 2015).

Certainly, one part of the increased health needs is caused by increased availability of health care and protection as well as the aging population and the increasing number of patients in the advanced stages of the disease. Hospitalization rate increased in the level of tertiary-care, which is probably the consequence of poor reorganization at lower levels of health care. Primary health care remained robust and bureaucratic. It relies on general practitioners who work in the primary health care institutions and are not the type of family doctors as it is common in most of Western countries. It seems that health authorities in most of former Yugoslav republics lost their chance to implement major reforms of the health systems in their countries. Stronger insistence on outpatient prescription and dispensing of medicines should be the long-term aim in developing countries as former Yugoslav republics, since this approach is widely considered as one of the main guardians of the whole healthcare system (Jakovljevic et al., 2015b).

## Ethics statement

Ethics Committee consideration and approval are not necessary in accordance with Good Research Practice guidelines, in retrospective studies observing national level data.

## Author contributions

All authors listed, have made substantial, direct and intellectual contribution to the work, and approved it for publication. AC and NZ developed research questions, designed the study, and prepared manuscript for this Data Report. DC and VS participated in the presentation and interpretation of the results.

### Conflict of interest statement

The authors declare that the research was conducted in the absence of any commercial or financial relationships that could be construed as a potential conflict of interest.

## References

[B1] Alkire B. C. Shrime M. G. Dare A. J. Vincent J. R. Meara J. G. (2015). The global economic consequences of selected surgical diseases: a modelling study. Lancet Glob. Health 3, 21–27. 10.1016/S2214-109X(15)70088-4 25926317 PMC4884437

[B2] Arcaya M. C. Arcaya A. L. Subramanian S. V. (2015). Inequalities in health: definitions, concepts, and theories. Glob Health Action 8:27106. 10.3402/gha.v8.27106 26112142 PMC4481045

[B3] Barnett A. G. Page K. Campbell M. Martin E. Rashleigh-Rolls R. Halton K.. (2013). The increased risks of death and extra lengths of hospital and ICU stay from hospital-acquired bloodstream infections: a case-control study. BMJ Open. 3:e003587. 10.1136/bmjopen-2013-003587 24176795 PMC3816236

[B4] Brunaud L. Reibel N. Ayav A. (2011). Pancreatic, endocrine and bariatric surgery: the role of robot-assisted approaches. J. Visc. Surg. 148, 47–53. 10.1016/j.jviscsurg.2011.05.006 21978931

[B5] Burnham C-A. D. Carroll K. C. (2013). Diagnosis of Clostridium difficile infection: an ongoing conundrum for clinicians and for clinical laboratories. Clin. Microbiol. Rev. 26, 604–630. 10.1128/CMR.00016-13 23824374 PMC3719497

[B6] Caruso S. Patriti A. Roviello F. De Franco L. Franceschini F. Coratti A.. (2016). Laparoscopic and robot-assisted gastrectomy for gastric cancer: current considerations. World J. Gastroenterol. 22, 5694–5717. 10.3748/wjg.v22.i25.5694 27433084 PMC4932206

[B7] Denny K. J. Cotta M. O. Parker S. L. Roberts J. A. Lipman J. (2016). The use and risks of antibiotics in critically ill patients. Expert Opin. Drug Saf. 15, 667–678. 10.1517/14740338.2016.1164690 26961691

[B8] Forster A. J. Stiell I. Wells G. Lee A. J. van Walraven C. (2003). The effect of hospital occupancy on emergency department length of stay and patient disposition. Acad. Emerg. Med. 10, 127–133. 10.1111/j.1553-2712.2003.tb00029.x 12574009

[B9] Jakovljevic M. (2013). Resource allocation strategies in Southeastern European health policy. Eur. J. Health Econ. 14, 153–159. 10.1007/s10198-012-0439-y 23143312

[B10] Jakovljevic M. (2015). BRIC's growing share of global health spending and their diverging pathways. Front. Public Health 3:135. 10.3389/fpubh.2015.00135 26000273 PMC4421927

[B11] Jakovljevic M. Laaser U. (2015). Population aging from 1950 to 2010 in seventeen transitional countries in the wider region of South Eastern Europe. South East. Eur. J. Public Health, 3 10.12908/SEEJPH-2014-42

[B12] Jakovljevic M. M. Ogura S. (2016). Health economics at the crossroads of centuries - from the past to the future. Front. Public Health 4:115. 10.3389/fpubh.2016.00115 27376055 PMC4899886

[B13] Jakovljevic M. Vukovic M. Chen C. C. Antunovic M. Dragojevic-Simic V. Velickovic-Radovanovic R.. (2016). Do health reforms impact cost consciousness of health care professionals? Results from a nation-wide survey in the Balkans. Balkan Med. J. 33, 8–17. 10.5152/balkanmedj.2015.15869 26966613 PMC4767315

[B14] Jakovljevic M. B. Vukovic M. Fontanesi J. (2015a). Life expectancy and health expenditure evolution in eastern Europe - DiD and DEA analysis. Expert Rev. Pharmacoecon. Outcomes Res. 17, 1–10. 10.1586/14737167.2016.1125293 26606654

[B15] Jakovljević M. Jovanović M. Lazić Z. Jakovljević V. ÄŘukić A. Veličković R. (2011). Current efforts and proposals to reduce healthcare costs in Serbia. Serbian J. Exp. Clin. Res. 12, 161–163. 10.5937/sjecr1104161J

[B16] Jakovljevic M. B. Djordjevic N. Jurisevic M. Jankovic S. (2015b). Evolution of the Serbian pharmaceutical market alongside socioeconomic transition. Expert Rev. Pharmacoecon. Outcomes Res. 15, 521–530. 10.1586/14737167.2015.1003044 25592856

[B17] Kandel C. E. Gill S. McCready J. Matelski J. Powis J. E. (2016). Reducing co-administration of proton pump inhibitors and antibiotics using a computerized order entry alert and prospective audit and feedback. BMC Infect. Dis. 16:355. 10.1186/s12879-016-1679-8 27449956 PMC4957393

[B18] Karam G. Chastre J. Wilcox M. H. Vincent J. L. (2016). Antibiotic strategies in the era of multidrug resistance. Crit Care 20, 136. 10.1186/s13054-016-1320-7 27329228 PMC4916531

[B19] Kovacević A. Dragojević-Simić V. Rancić N. Jurisević M. Gutzwiller F. S. Matter-Walstra K.. (2015). End-of-life costs of medical care for advanced stage cancer patients. Vojnosanit. Pregl. 72, 334–341. 10.2298/VSP1504334K 26040179

[B20] Kramer A. A. Zimmerman J. E. (2010). A predictive model for the early identification of patients at risk for a prolonged intensive care unit length of stay. BMC Med. Inform. Decisi. Mak. 10:27. 10.1186/1472-6947-10-27 20465830 PMC2876991

[B21] Mackenbach J. Stirbu I. Roskam A. J. Schaap M. Menvielle G. Leinsalu M. (2008). European union working group on socioeconomic inequalities in health. socioeconomic inequalities in health in 22 European countries. N.Engl. J. Med. 358, 2468–2481. 10.1056/NEJMsa0707519 18525043

[B22] Mathur H. Rea M. C. Cotter P. D. Ross R. P. Hill C. (2014). The potential for emerging therapeutic options for Clostridium difficile infection. Gut Microbes 5, 696–710. 10.4161/19490976.2014.983768 25564777 PMC4615897

[B23] Ogura S. Jakovljevic M. (2014). Health financing constrained by population agingan opportunity to learn from Japanese experience. Ser. J. Exp. Clin. Res. 15, 175–181. 10.2478/sjecr-2014-0022

[B24] Petrusic T. Jakovljevic M. (2015). Budget impact of publicly reimbursed prescription medicines in the Republic of Srpska. Front. Public Health 3:213. 10.3389/fpubh.2015.00213 26442240 PMC4564658

[B25] Sganga G. Tascini C. Sozio E. Carlini M. Chirletti P. Cortese F.. (2016). Focus on the prophylaxis, epidemiology and therapy of methicillin-resistant *Staphylococcus aureus* surgical site infections and a position paper on associated risk factors: the perspective of an Italian group of surgeons. World J. Emerg. Surg. 11:26. 10.1186/s13017-016-0086-1 27307786 PMC4908758

[B26] Shrime M. G. Dare A. Alkire B. C. Meara J. G. (2016). A global country-level comparison of the financial burden of surgery. Br. J. Surg. 103, 1453–1461. 10.1002/bjs.10249 27428044

[B27] Shrime M. Dare A. Alkire B. O'Neill K. Meara J. (2015). Catastrophic expenditure to pay for surgery: a global estimate. Lancet Glob. Health 3, 38–44. 10.1016/S2214-109X(15)70085-9 25926319 PMC4428601

[B28] Getzen T. (1992). Population aging and the growth of health expenditures. J. Gerontol. 47:98–104. 10.1093/geronj/47.3.S98 1573213

[B29] Udy A. A. Roberts J. A. Lipman J. (2013). Clinical implications of antibiotic pharmacokinetic principles in the critically. Intensive Care Med. 39, 2070–2082. 10.1007/s00134-013-3088-4 24045886

[B30] UN, World Population Ageing (2013). United Nations Department of Economic and Social Affairs Population, Division, The World Population, Ageing, New York, NY: United Nations (2013). Available online at: http://www.un.org/en/development/desa/population/publications/ageing/WorldPopulationAgeingReport2013.shtml (accessed October 6, 16).

[B31] Vincent J-L. Bassetti M. François B. Karam G. Chastre J. Torres A.. (2016). Advances in antibiotic therapy in the critically ill. Crit Care 20, 133. 10.1186/s13054-016-1285-6 27184564 PMC4869332

[B32] WHO (2015). World Health Organization, European Health for All database (HFA-DB). Available online at: http://www.euro.who.int/en/data-and-evidence/databases/european-health-for-all-database-hfa-db (Accessed October 6, 16).

[B33] Ye Y. L. Yuan X. X. Chen M. K. Dai Y. P. Qin Z. K. Zheng F. F. (2016). Management of adrenal incidentaloma: the role of adrenalectomy may be underestimated. BMC Surg. 16:41. 10.1186/s12893-016-0154-1 27278528 PMC4898397

[B34] Yin J. Lurås H. Hagen T. P. Dahl F. A. (2013). The effect of activity-based financing on hospital length of stay for elderly patients suffering from heart diseases in Norway. BMC Health Serv. Res. 13:172. 10.1186/1472-6963-13-172 23651910 PMC3651263

[B35] Zhou J.-Y. Xin C. Mou Y.-P. Xu X.-W. Zhang M.-Z, Zhou, Y. C.. (2016). Robotic versus laparoscopic distal pancreatectomy: a meta-analysis of short-term outcomes. PLoS ONE 11:e0151189. 10.1371/journal.pone.0151189 26974961 PMC4790929

